# Glucagon-Like Peptide-1 as Predictor of Body Mass Index and Dentate Gyrus Neurogenesis: Neuroplasticity and the Metabolic Milieu

**DOI:** 10.1155/2014/917981

**Published:** 2014-11-23

**Authors:** Jeremy D. Coplan, Shariful Syed, Tarique D. Perera, Sasha L. Fulton, Mary Ann Banerji, Andrew J. Dwork, John G. Kral

**Affiliations:** ^1^Department of Psychiatry & Behavioral Sciences, Division of Neuropsychopharmacology, State University of New York, Downstate Medical Center (SUNY DMC), Brooklyn, NY 11203, USA; ^2^Downstate College of Medicine, SUNY DMC, Brooklyn, NY 11203, USA; ^3^New York State Psychiatric Institute, New York, NY 10032, USA; ^4^Division of Endocrinology, Department of Internal Medicine, SUNY DMC, Brooklyn, NY 11203, USA; ^5^Department of Molecular Imaging and Neuropathology, New York State Psychiatric Institute, New York, NY 10032, USA; ^6^Departments of Psychiatry and Pathology and Cell Biology, College of Physicians and Surgeons of Columbia University, New York, NY 10032, USA; ^7^Departments of Surgery and Internal Medicine, SUNY DMC, Brooklyn, NY 11203, USA

## Abstract

Glucagon-like peptide-1 (GLP-1) regulates carbohydrate metabolism and promotes neurogenesis. We reported an inverse correlation between adult body mass and neurogenesis in nonhuman primates. Here we examine relationships between physiological levels of the neurotrophic incretin, plasma GLP-1 (pGLP-1), and body mass index (BMI) in adolescence to adult neurogenesis and associations with a diabesity diathesis and infant stress. Morphometry, fasting pGLP-1, insulin resistance, and lipid profiles were measured in early adolescence in 10 stressed and 4 unstressed male bonnet macaques. As adults, dentate gyrus neurogenesis was assessed by doublecortin staining. High pGLP-1, low body weight, and low central adiposity, yet peripheral insulin resistance and high plasma lipids, during adolescence were associated with relatively high adult neurogenesis rates. High pGLP-1 also predicted low body weight with, paradoxically, insulin resistance and high plasma lipids. No rearing effects for neurogenesis rates were observed. We replicated an inverse relationship between BMI and neurogenesis. Adolescent pGLP-1 directly predicted adult neurogenesis. Two divergent processes relevant to human diabesity emerge—high BMI, low pGLP-1, and low neurogenesis and low BMI, high pGLP-1, high neurogenesis, insulin resistance, and lipid elevations. Diabesity markers putatively reflect high nutrient levels necessary for neurogenesis at the expense of peripheral tissues.

## 1. Introduction

The precedent for a relationship between metabolic processes and neuroprotection is ontogenetically evident in the invertebrate,* C. elegans*, involving DAF2-mediated gene expression, a homolog of mammalian insulin receptor expression [1]. DAF-2 tightly regulates fatty acid and amino acid metabolism [2]. In light of the evolutionary conservation of the insulin/insulin-like growth factor 1 (IGF-1) signaling (IIS) pathway, studies extrapolated from* C. elegans* shed light on its functions and regulation in higher organisms, including nonhuman primates and humans [3]. For instance, downregulation of the insulin/IGF-1 signaling pathway has been demonstrated to induce diapause in* C. elegans*, a state associated with neuronal loss [4].

GLP-1 is an insulinotropic incretin produced by enteroendocrine L cells found throughout the small intestine [5]. GLP-1 and its receptor system have been shown to affect central satiety processes and feeding behavior [6]. Administration of an insulinotropic GLP-1 agonist, exendin (ex-4), inhibits food intake, reducing body mass [7] while stimulating adult dentate gyrus neurogenesis [8]. We have previously shown an inverse correlation between body mass and dentate gyrus neurogenesis and body mass and expression of the antimitotic gene factor, Bcl-2 (B-cell CLL/lymphoma2) in bonnet macaques [9]. Moreover, in humans, using magnetic resonance spectroscopy, we have noted an inverse relationship between BMI and right hippocampal N-acetyl-aspartate (NAA) concentrations, a molecular marker of neuronal viability [10], although this effect, because of limitations in anatomical resolution, cannot be specifically attributed to the dentate gyrus.

We have previously reported persistent increases in plasma GLP-1 (pGLP-1) concentrations following early life stress (ELS) induced by variable foraging demand (VFD) rearing compared to unstressed control (USC) monkeys [11]. The VFD model of ELS has been shown to induce a multitude of effects including subsequent reductions in left hippocampal volume and anxious behavior [12], elevations of cisternal cerebrospinal fluid serotonin metabolites [13], amygdala hypertrophy [14], decreased white matter integrity of the anterior limb of the internal capsule [15], and insulin resistance [11]. Despite increases in pGLP-1, VFD rearing was associated with impaired glucose disposal versus nonstressed controls. We speculated that GLP-1 resistance may be present, or “compensatory” insulin resistance (IR) had emerged following VFD rearing, a possibility that has been raised in insulin-resistant South Asian humans [16]. However, GLP-1 was not examined in the context of current BMI and future neurogenesis.

Given the current obesity epidemic [17] and associated metabolic morbidity and mortality [18], further investigation of the context of the inverse relationship between neurogenesis and body metabolism is warranted. Our specific hypothesis was that GLP-1 may play a critical dual role in regulating systemic energy balance in setting the stage for future hippocampal neurogenesis. We therefore build on our previous study [9] by comprehensively examining the metabolic milieu that fosters or hinders long-term neuroplasticity. An additional goal was to examine potential correlates relevant to the human diabesity epidemic.

## 2. Methods

### 2.1. Subjects

Among 14 male bonnet macaques (*Macaca radiata*) 10 had been reared under variable foraging demand (VFD) whereas 4 were reared under unstressed control (USC) conditions [11]. At an age that corresponds to human adolescence, pGLP-1, insulin-related measures, lipid profiles, and morphometrics were obtained. Data for insulin measures were only available on 11 subjects (eight VFD and three USC). Data on the euglycemic insulin clamp procedure [11] were only available on eight subjects (five VFD and three USC). There were no statistical differences between the groups in age (VFD: 51 ± 8 versus USC: 49 ± 6 lunar months; *P* = 0.62) or weight (VFD: 4.1 ± 0.8 kg; USC: 4.4 ± 0.8; *P* = 0.50) when considering the entire sample. Neurogenesis studies were performed following sacrifice in adulthood. At sacrifice, VFD-reared subjects were less than one year younger than USC, but nevertheless statistically significantly younger than USC (VFD: 99 ± 5 lunar months, USC: 108 ± 3 lunar months; *P* = 0.017) with negligible weight differences (VFD: 10.1 ± 2.1 kg, USC: 11.1 ± 2.5; *P* = 0.44 kg).

### 2.2. Rearing

Mother-infant dyads were group-housed in pens of 5–7 dyads each and stabilized for at least four weeks prior to VFD onset. After 2 months of infant age, while still exclusively nursing, dyads were subjected to a standard VFD procedure (early life stress, ELS) that involved 8 alternating 2-week blocks in which an adequate supply of maternal food was more difficult to access (high foraging demand) or was more readily obtained (low foraging demand) [19]. The control condition was 16 continuous weeks of non-VFD (unstressed control (USC)). Available food quantities were the same under both conditions and there were no differences in body weight between VFD and USC mothers or infants. However, the unpredictability of foraging conditions prevented VFD mothers from adequately addressing the emotional needs of their infants which was stressful for both mother and infant. Achieving the ELS paradigm therefore occurs through the disruption of normative patterns of maternal rearing and infant attachment [20]. After weaning there were no differences in any of the animals' access to standard chow, supplemented with fresh fruit throughout the life cycle.

### 2.3. Morphometry

During anesthesia for blood sampling, body weight in kilograms, body mass index (BMI), crown rump length (Crl), and sagittal abdominal diameter (SAD) were measured. Crl is the length in centimeters from the vertex of the head to the base of the tail. SAD, a measure of abdominal or “visceral” adipose tissue volume, is the distance from the surface of the examination table to the top of the abdomen at its highest point with the animal lying flat on its back. Abdominal (waist) circumference is the largest distance in centimeters around the abdomen at the level of the iliac crests. Measurements were performed by investigators blinded to rearing condition. A modified monkey BMI was calculated as mass in kilograms divided by the square of the Crl in meters.

### 2.4. Blood Chemistry

After an overnight fast (food withdrawn at 1600 h, water* ad libitum*), monkeys entered individual carrying cages between 0800 and 1100 h and were moved to squeeze cages where they were anesthetized with ketamine (10–15 mg/kg). Antecubital or femoral venous blood was immediately placed on ice, centrifuged, and stored at −80°C within one hour, as described previously [11]. Samples were analyzed for plasma glucose, triglycerides (TGs), very low density lipoprotein (VLDL), HDL, LDL, and total cholesterol by routine methods at the University Hospital of Brooklyn Clinical Chemistry Laboratory (Brooklyn, NY). Plasma insulin and glucagon-like peptide-1 (pGLP-1) were analyzed using the Human Endocrine Lincoplex kit (Linco Diagnostic Services). Insulin resistance (IR) was calculated as insulin/glucose ratio (IGR) and homeostatic model assessment of insulin resistance (HOMA-IR) computed from the following formula:

(1) $$\begin{array}{c} \text{HOMA-IR}=\frac{\text{Insulin}\times \text{Glucose}}{22.5}, \end{array}$$

first described by Turner et al. [19], and the euglycemic insulin clamp, a highly sensitive procedure for calculating insulin resistance in clinical studies described in detail in [11].

### 2.5. Neurohistochemistry

**Figure 1:**
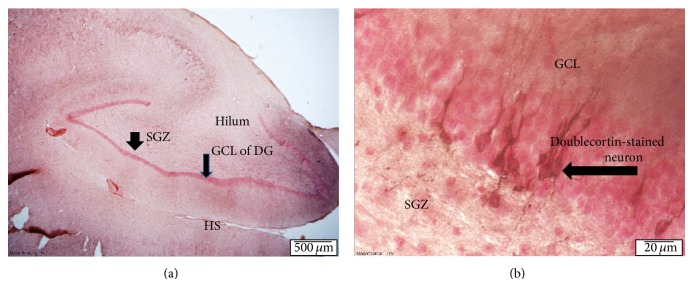
Doublecortin staining of neurogenesis in the dentate gyrus of the hippocampus. In (a), we present the dentate gyrus (DG) of the left hippocampus at 2x magnification (see scale) and (b) is at 40x magnification of the dentate gyrus (see scale). Doublecortin stained neurons can be seen in red extending from the subgranular zone (SGZ) into the granule cell layer (GCL) with staining of tertiary dendrites. DG is dentate gyrus. SGZ is subgranular zone. HS is hippocampal sulcus. All sections are taken from the anterior hippocampus.

Subjects were anesthetized to a surgical depth with sodium pentothal and transcardially perfused with saline and formalin [21]. The brains were removed and postfixed in 4% paraformaldehyde for immunohistochemical staining and analysis. The anterior left hippocampus was cut into 40 *μ*m sections and every 40th section was immunostained to detect cells labeled with the immature neuronal marker doublecortin (DCX) using the monoclonal primary antibody anti-DCX (1 : 3000; Santa Cruz) (see Figure 1). The secondary antibody was biotinylated horse anti-mouse or rabbit anti-goat IgG (1 : 200; Vector Laboratories) visualized with avidin-biotin complex solution (Vector) and diaminobenzidine (DAB; Sigma). For each animal, the density of DCX-labeled cells per mm^3^ of the subgranular zone (SGZ) was measured microscopically. Two independent raters, masked to treatment condition (interrater reliability > 0.95) counted all unambiguously DCX-labeled cells in the SGZ of the dentate gyrus (defined as a two-cell-body-wide zone on either side of the border of the granule cell layer) using a 40x objective.

### 2.6. Procedures

All animal procedures were approved by the SUNY Downstate Medical Center Institutional Animal Care and Use Committee (IACUC).

### 2.7. Statistics

Adult doublecortin counts, adolescent BMI, and adolescent pGLP-1 levels were assessed for outliers and tested for normality of distribution.

#### 2.7.1. Predictor Variables of Adult Doublecortin Counts (See Table 1)

Doublecortin counts served as the dependent variable in a General Linear Model (GLM; Statistica 12.0). Primary adolescent predictor variables included pGLP-1 and BMI and as secondary predictors and the morphometric, metabolic, and lipid measures outlined above, as well as adult age and weight at sacrifice. VFD served as an independent categorical variable. The primary hypotheses were that when controlled for rearing status, adolescent pGLP-1 levels would positively predict adult neurogenesis rates and adolescent BMI would inversely predict adult neurogenesis rates. The Partial Eta Squared was used to evaluate effect sizes of significant results where relevant [22]. Adolescent age and/or adult age were used as covariates when proven to be significant predictors of adult doublecortin counts. Based on the considerations described below, post hoc Spearman's correlations for adult doublecortin and predictor variables were applied to the combined sample for nonparametric validation of GLM results. Insulin levels, insulin/glucose ratio, and HOMA were only available on 11 subjects (8 VFD and 3 USC). Since the insulin euglycemic clamp M-rate (index of amount of glucose disposed = insulin sensitivity) data (only available for *n* = 5 VFD and *n* = 3 USC) were repeated measures for 30-, 60-, and 90-minute time points, we performed an analysis of variance with repeated measures (GLM with repeated measures) with doublecortin as a predictor variable and rearing as a covariate with post hoc univariate analyses for each time point.

#### 2.7.2. Variables Predictive of pGLP-1

Since adolescent pGLP-1 was hypothesized to predict adult doublecortin, we wished to examine continuous variables that predicted pGLP-1 as a dependent variable. Since distributions were normal, Pearson correlations examined whether the associations between pGLP-1 as the dependent variable and adolescent morphometric, metabolic, and lipid measures and adult body mass as the predictor variable varied as a function of ELS. Differences in “rho” were determined for Pearson's correlations within-VFD and within-USC and all study subjects were then combined. Where significant between-groups differences in *r* values were observed, we performed a GLM analysis with GLP-1 as the dependent variable, rearing group as the independent categorical variable, and the candidate metabolic variable serving as predictor (plasma insulin, insulin/glucose ratio, and HOMA were only available on *n* = 8 VFD and on *n* = 3 USC). The GLM was then configured as a factorial ANOVA where rearing effects, predictor variable effects, and their interactions were assessed. A significant interactive effect would imply that a given metabolic variables' relationship to pGLP-1 differed as a function of early life stress.

#### 2.7.3. Concordance Analyses

Should a high number of interrelated correlations be observed, we deemed it important to examine the significance of concordance of multiple dependent variables. Significance would imply a coherent biological system entailing multiple peripheral and central processes culminating in adult neurogenesis rates. Without claiming causality, we invoke the work in the invertebrate,* C. elegans*, indicating that the pivotal variable would be GLP-1, an incretin whose role in governing metabolism and neuronal proliferation has been conserved in mammalian physiology [23].

We therefore ran Friedman's nonparametric ANOVA for multiple dependent variables, generating the Kendall coefficient of concordance, which would serve to validate the interdependence of, in adolescence, pGLP-1, BMI, LDL, and insulin/glucose ratio and their longitudinal concordance with adult neurogenesis. The analysis first omitted (*N* = 14) and then included (*N* = 11) IR measures. The analysis was rerun only in VFD, without (*N* = 10) and with (*N* = 8) IR measures to ascertain that concordance effects were at least specifically evident in VFD-reared subjects.

Significance was determined as *P* ≤ 0.05, two-tailed.

## 3. Results

**Figure 2:**
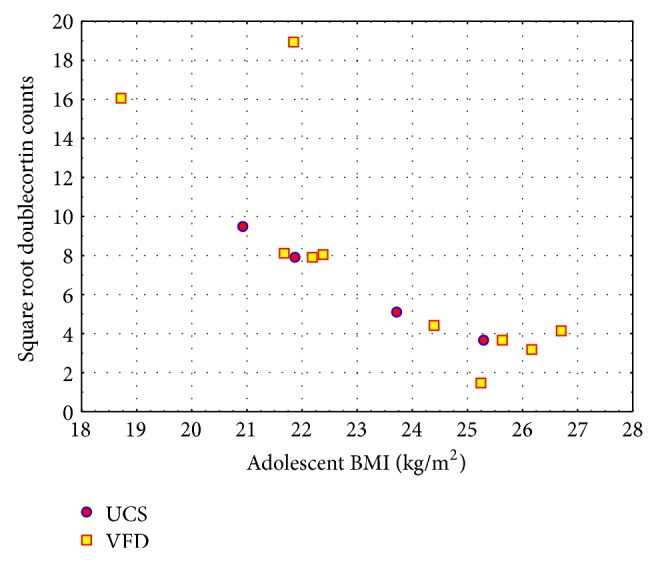
Prediction of adult square root doublecortin by adolescent body mass index. There was no rearing effect for SQRT doublecortin [*F* = 1.18; *P* = 0.30] whereas adolescent body mass index predicted adult SQRT doublecortin [*F*1,11 = 22.48; *P* = 0.0006]. Post hoc Spearman *r* = −0.88; *N* = 14; *P* = 0.00002. Partial *η* ^2^ = 0.67 (effect size ≥ 0.14 = large effect size). Red circles: USC (unstressed controls); blue circles: VFD.

Adolescent pGLP-1 and adolescent BMI were normally distributed. Since the doublecortin counts did not satisfy conditions of normality (Lilliefors' test *P* < 0.01), we square-rooted (SQRT) doublecortin counts, which effectively normalized the values. There were no outliers although there were two subjects with relatively high SQRT doublecortin values (see Figure 2). We therefore adopted the more conservative Spearman's nonparametric correlation for validation of predictors of SQRT doublecortin. Neither adolescent age [*r*_sp_ = −0.26; *N* = 14; *P* = 0.36] nor adult age [*r*_sp_ = −0.32; *N* = 14, *P* = 0.26] were significant predictors of SQRT doublecortin counts and were therefore not used as covariates (Table 1).

### 3.1. Predictors of SQRT Doublecortin Counts

Controlling for rearing condition, adolescent pGLP-1, as hypothesized, positively predicted adult SQRT doublecortin (partial *η* ^2^ = 0.33), an effect size twice that required for a “large” effect size (0.14) and confirmed by nonparametric Spearman correlations. Adolescent BMI, consistent with the primary hypothesis of the study, inversely predicted adult neurogenesis (partial *η* ^2^ = 0.67), an effect size four times that required for a large effect size and confirmed by nonparametric analyses (see Figure 2). Additional adolescent morphometric predictor variables, including abdominal circumference, sagittal abdominal diameter, and body mass, but not adolescent age and crown rump length, also inversely predicted adult neurogenesis. Interestingly, controlling for rearing condition and confirmed nonparametrically, analyses supported a positive relationship between adult neurogenesis and adolescent plasma insulin, insulin/glucose ratio, and HOMA-IR, all indicators of insulin resistance (although insulin/glucose ratio was only a marginal predictor of SQRT doublecortin when controlling for rearing condition). Regarding adolescent lipid profiles, both LDL and total cholesterol positively predicted adult neurogenesis. Although adult age was a significant inverse predictor of neurogenesis when controlling for rearing, this relationship did not remain (even marginally) significant when tested nonparametrically; adult weight did not predict neurogenesis. Rearing condition did not predict SQRT doublecortin in any of the analyses. There were no relationships of SQRT doublecortin to M-rate measures derived from the euglycemic clamp procedure.

### 3.2. Predictors of pGLP-1 and Rearing Group Correlational Differences (Table 2)

Adolescent BMI (kg/m^2^) was inversely correlated with pGLP-1 in VFD subjects and when combining all subjects with no “*r*” value difference between rearing groups. No relationship between CRL (cm) and pGLP-1 was noted. For abdominal circumference (cm), a markedly inverse correlation with pGLP-1 was noted in VFD subjects but the same correlation in non-VFD subjects was nonsignificantly positive, leading to a significant “*r*” difference between rearing groups (see Figure 2). GLM revealed a significant rearing group ∗ abdominal circumference interaction in the prediction of pGLP-1 [*F*_(1,10)_ = 20.11; *P* = 0.0012, partial *η* ^2^ = 0.66] (an effect size almost seven factors greater than the minimum required for a “large” effect size). When adjusting for abdominal circumference, pGLP-1 concentrations (pmol/L) were significantly higher in VFD versus non-USC [VFD mean (SE) = 85.00 (5.45) versus USC mean (SE) = 45.68 (8.61); *F*_(1,10)_ = 21.43; *P* = 0.0009; partial *η* ^2^ = 0.66]. An inverse relationship between pGLP-1 and sagittal abdominal diameter was noted only in the combined group.

Consistent with its role as an incretin, positive correlations were noted between pGLP-1 and insulin. The incretin effect was significant in VFD subjects and the combined group and directionally similar in USC. But positive correlations were also observed for the insulin/glucose ratio, HOMA, and M-rate at 60 (but not 30 and 90) minutes of the euglycemic insulin clamp procedure in all subjects combined, indicating paradoxical IR with increasing pGLP-1. No correlations were noted between pGLP-1 and plasma glucose. LDL was positively associated with pGLP-1 in VFD and in all subjects together. Total cholesterol was positively associated with pGLP-1 in VFD subjects but the association was inverse in USC subjects, reflected in a significant “*r*” difference with no significant effects in the GLM. Similarly there were positive associations between pGLP-1 and VLDL in VFD subjects and the combined group. Adolescent pGLP-1 was inversely associated with adult body mass in VFD but positively associated with adult body mass in USC, leading to a significant difference in the “*r*” value. GLM revealed a significant rearing group ∗ adult body mass interaction in the prediction of pGLP-1 [*F*_(1,10)_ = 6.29; *P* = 0.031, partial *η* ^2^ = 0.38, 2.5x greater than the cutoff for a large effect size] (see Figure 4) whereas when adjusting for adult body mass, pGLP-1 concentrations were significantly increased in VFD versus USC subjects [VFD mean (SE) = 85.00 (11.13) versus USC mean (SE) = 45.68 (17.60); *F*_(1,10)_ = 7.41, *P* = 0.021]. The euglycemic clamp procedure, consistent with the Pearson correlations, in univariate analyses, controlling for rearing, revealed that, at the 60-minute (but not 30 or 90) time point, pGLP-1 was inversely related to M-rate, suggesting that high pGLP-1 was related to insulin resistance [*F*_(1,4)_ = 15.34; *P* = 0.011; partial *η* ^2^ = 0.75]. VFD rearing was not associated with any group differences on independent *t*-tests except for lower triglycerides in VFD as adolescents [VFD mean (sd) = 13.60 (3.86)  *N* = 10 versus USC mean (sd) = 24.00 (5.83)  *N* = 4; *t*-value = 3.96; *P* = 0.019].

### 3.3. Adolescent BMI as Predictor of Insulin Resistance Markers and Lipid Profiles

In order to better understand the relationship between the paradoxically positive relationship between adult neurogenesis and adolescent pGLP-1 to both insulin resistance and plasma lipids and inverse relationship to body mass index, we examined the relationship of body mass index to markers of insulin resistance and plasma lipids. Paradoxical relationships were evident where relatively high BMI predicted low insulin [*r* = −0.88, *N* = 11, *P* < 0.001], low insulin/glucose ratio [*r* = −0.87, *N* = 11, *P* < 0.001], and HOMA-IR [*r* = −0.85, *N* = 11, *P* = 0.001], but not plasma glucose [*r* = 0.33, *N* = 11, *P* = 0.24]. There was no relationship between BMI and plasma triglycerides [*r* = −0.12, *N* = 14, *P* = 0.68] or HDL [*r* = −0.47, *N* = 14, *P* = 0.087] but there were paradoxically inverse relationships with LDL [*r* = −0.75, *N* = 14, *P* = 0.002], cholesterol [*r* = −0.75, *N* = 14, *P* = 0.002], and VLDL [*r* = −0.54, *N* = 14, *P* = 0.044]. Thus, low BMI paradoxically predicted insulin resistance and elevated lipids. BMI did not predict M-rate derived from the euglycemic insulin procedure at the 30-, 60-, or 90-minute time points.

### 3.4. Analysis of Concordance

When including pGLP-1, BMI, LDL, and SQRT doublecortin, the Friedman ANOVA revealed a Chi Square [*N* = 14, df = 3] = 36.25 *P* < 0.00001 with a Kendall coefficient of concordance = 0.86. This effect appeared numerically stronger with inclusion of insulin/glucose ratio despite loss of three subjects [Friedman ANOVA Chi Square (*N* = 11, df = 4) = 41.81, *P* = < 0.00001, and Kendall coefficient of concordance = 0.95].

When considering only VFD, concordance effects were essentially identically significant.

## 4. Discussion

We confirmed our primary hypothesis that adolescent pGLP-1 levels are positively associated with adult neurogenesis rates in nonhuman primates and replicated the inverse relation between adult neurogenesis and BMI [7], albeit the latter measured in adolescence. Both of these effects were present when controlling for rearing and when applying conservative nonparametric analyses. Large effect sizes were present, more than twofold for the adolescent pGLP-1/adult neurogenesis relationship and fourfold for the adolescent BMI/adult neurogenesis relationship, when controlling for rearing effects. Age was not a significant predictor of neurogenesis when nonparametric analyses were applied. No rearing effects were observed for doublecortin counts. Morphometric measures associated with central adiposity, such as sagittal abdominal diameter and abdominal circumference, were also inversely related to neurogenesis counts, suggesting that adolescent vulnerability to a nonhuman primate form of metabolic syndrome was associated with relatively low neurogenesis. The current findings are generally consistent with findings in rodents indicating that pGLP-1 is associated with increased neurogenesis [8, 24, 25]. Consistent with* C. elegans* physiology, whereas in diapause there is downregulation of the insulin/IGF-1 signaling pathway and neuronal loss [1], we demonstrate that relative increases of the incretin, GLP-1, are associated with a relative increase in neurogenesis.

Curiously, however, relatively high neurogenesis was positively associated with adolescent biomarkers indicative of insulin resistance, including plasma insulin, insulin/glucose ratio, and HOMA-IR. Of note, glucose levels are not related to neurogenesis, suggesting that the expected glucose elevations associated with increasing insulin resistance were not observed, with putative utilization by relatively high neurogenesis. These data suggest that adolescent insulin resistance paradoxically is present concomitant with an inverse relationship between pGLP-1 and adolescent BMI and subsequently positively associated with neurogenesis in adulthood (Figure 5), whereas the literature indicates that drugs that reduce IR are associated with an increase in neurogenesis. This raises the possibility of a complex, ostensibly paradoxical physiological metabolic developmental pattern either orchestrated or compensated by pGLP-1 setting the stage for future neurogenesis. The data thus suggest two divergent conditions or stages relevant to human diabesity—high BMI associated with low pGLP-1 (“resistance”) and impaired neurogenesis and high plasma GLP-1 associated with high neurogenesis and low BMI, in the presence of the insulin resistance and dyslipidemia of diabesity. Diabesity markers putatively reflect maintenance or partitioning of nutrients for neurogenesis at the expense of peripheral tissues. Adult hippocampal neurogenesis, it is noted, occurs within a specific microenvironment, where glucose (and lipid) supply to mesenchymal stem cells is vital [26]. In this model, provision of glucose and lipids comprise essential metabolic substrates in the maintenance of the relatively high levels of neurogenesis observed in relatively low BMI macaques. The observation of a highly significant Kendall coefficient of concordance including the above variables suggests a coherent and coordinated response in this process.

**Figure 3:**
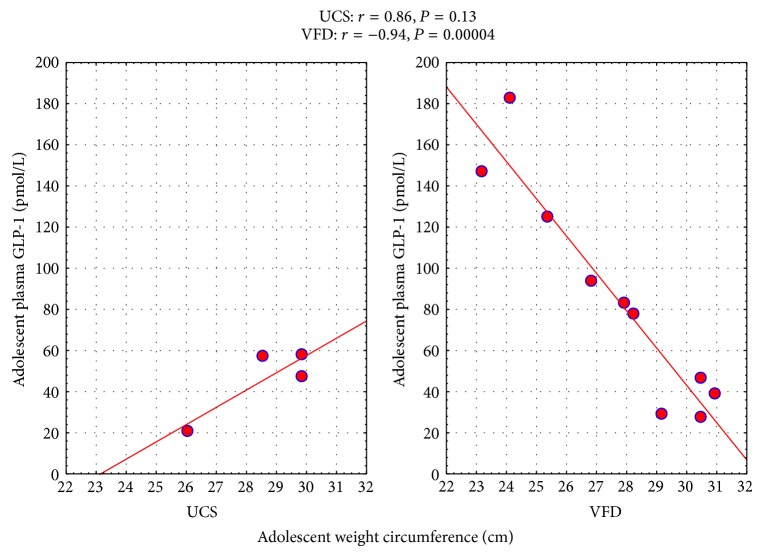
Relationship of adolescent pGLP-1 to adolescent waist circumference as a function of early life stress. For abdominal circumference (cm), a markedly inverse correlation with pGLP-1 was noted in VFD subjects but the same correlation in non-VFD subjects was nonsignificantly positive, leading to a significant “*r*” difference between rearing groups. GLM revealed a significant rearing group ∗ abdominal circumference interaction in the prediction of pGLP-1 [*F*_(1,10)_ = 20.11; *P* = 0.0012]. When adjusting for abdominal circumference, pGLP-1 concentrations (pmol/L) were significantly increased in VFD versus non-VFD subjects [VFD mean (SE) = 85.00 (5.45) versus non-VFD mean (SE) = 45.68 (8.61); *F*(1,10) = 21.43; *P* = 0.0009].

**Figure 4:**
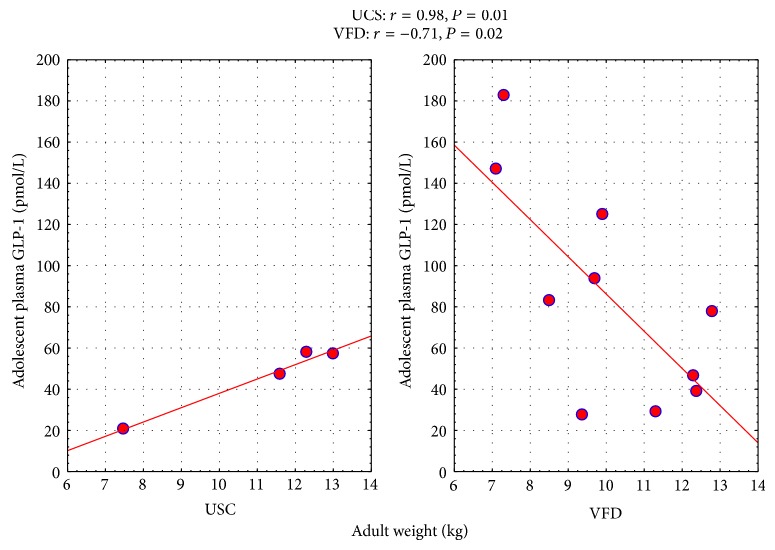
Relationship of adolescent pGLP-1 to adult body mass as a function of early life stress. Adolescent pGLP-1 was inversely associated with adult body mass in VFD but positively associated with adult body mass in non-VFD, leading to a significant difference in the “*r*” value. GLM revealed a significant rearing group ∗ adult body mass interaction in the prediction of pGLP-1 [*F*_(1,10)_ = 6.29; *P* = 0.031] whereas, when adjusting for adult body mass, pGLP-1 concentrations were significantly increased in VFD versus non-VFD subjects [VFD mean (SE) = 85.00 (11.13) versus non-VFD mean (SE) = 45.68 (17.60); *F*(1,10) = 7.41; *P* = 0.021].

**Figure 5:**
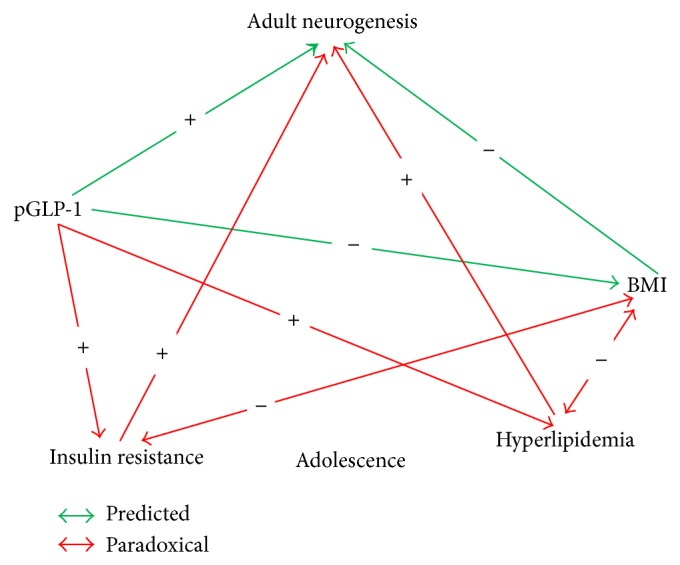
Schema of predicted and paradoxical associations of adolescent metabolic markers in the prediction of adult neurogenesis. Green lines indicate correlations that are observed to occur in the predicted direction whereas red lines are predictions that are observed to be opposite to the predicted direction. The significant Kendall coefficient of concordance for multiple dependent variables determined by the Friedman ANOVA (see text under results) raises the possibility of a complex, ostensibly paradoxical physiological metabolic pattern, which sets the stage for future neurogenesis, whereby glucose and lipids provide vital metabolic substrates in the maintenance of the relatively high levels of neurogenesis observed in relatively low BMI macaques.

pGLP-1, consonant with its incretin effect, was inversely associated with BMI, abdominal circumference, and sagittal abdominal diameter in the combined group. However, with respect to abdominal circumference, the inverse relationship observed in the VFD was not present in the USC, leading to a significant rearing group x abdominal circumference interaction, putatively indicating an evolving dysmetabolic diathesis. However, these interactive group differences may be artifactually related to the significantly elevated pGLP-1 in VFD when controlling for abdominal circumference (see Figure 3). Elevations of pGLP-1 above the cut-off 70 pmol/L, restricted to the VFD group, are associated with relatively low abdominal circumference (see Figure 3). pGLP-1 and insulin were positively correlated in all subjects. However, confirmatory of the previous observations involving adult neurogenesis, pGLP-1, directly predicts markers of insulin resistance without evident rearing effects. These observations conflict with a body of literature that incretins promote insulin sensitivity [27, 28], although not studied developmentally. We have previously shown higher GLP-1 levels concurrent with insulin resistance after VFD rearing positing a compensatory role of GLP-1 [11]. The aforementioned clinical studies examined the effects of exogenous GLP-1 agonists in adult diabetics whereas the current data examines correlates of pGLP-1 at ambient physiological doses in a bonnet macaque sample of which a substantial portion is VFD-reared. Curiously, there is a paradoxically positive relationship between pGLP-1 and LDL, total cholesterol and VLDL in all subjects, only significant in VFD. Exogenously administered pGLP-1 effectively abolishes the postprandial rise in triglyceride concentrations and lowers levels of nonesterified fatty acids in humans [29], an antidyslipidemic effect. The relationship of pGLP-1 to lipids in the current study is evidently more complex reflecting developmental changes where availability of lipids is secured, at least in part, for dentate gyrus neurogenesis, as reflected by the positive relationship between adolescent plasma lipids and adult doublecortin counts.

Regarding the relationship between adolescent pGLP-1 and adult body weight, there is a rearing group x adult body mass interaction whereby the expected inverse relationship is observed in the VFD subjects, whereas a positive relationship is observed in USC between adolescent body mass and adolescent pGLP-1. The interactive group effects have to be viewed with caution, given the low *N* (4 subjects) in the USC subjects. However, the effect size of the interactive effect is > 2.5-fold greater than the level required to be designated as a large effect size.

Although early life stress appears to influence the metabolic effects of pGLP-1, we fail to observe a rearing effect on adult neurogenesis rates, which is in contrast to a previous study in a small sample showing reductions of neurogenesis in VFD-reared subjects compared to controls [9]. Although we previously reported elevated pGLP-1 in VFD versus non-VFD subjects [11], the sample size in that study was large compared to the current study.

Limitations of the study include a relatively small number of USC subjects and the cross-sectional design. However, the number of experimental subjects provided sufficient power to detect rearing effects. Neurogenesis was measured in adulthood whereas pGLP-1, morphometric measures, insulin resistance, and lipid profiles were determined in early adolescence. However, the high number of adolescent findings concordant with adult neurogenesis provides coherent insight into the metabolic antecedents of later neuroplasticity. Although the adolescent measures are predictive of adult neurogenesis, the findings are correlational in nature and cannot infer causal mechanisms. However, the proximate role of GLP-1 is supported by the central role DAF2-mediated gene expression plays in controlling diapause in* C. elegans*, a primitive homolog of mammalian insulin receptor expression [1]. Another limitation is the lack of a clear explanation for the inverse relationship between BMI and neurogenesis. Certainly, high versus low body mass not only facilitates preferential access to food resources, but also demands increased daily caloric intake. Thus, the large caloric intake required to maintain a large body mass may necessitate a compensatory reduction in neurogenesis and enhancement of insulin sensitivity, shunting available nutrients for investment in peripheral tissues. By contrast, low body mass animals require less daily caloric intake and freeing up nutrients through peripheral insulin resistance for investment in high neurogenesis. Low versus high body mass may therefore represent divergent adaptive phenotypes. However, the question remains what the adaptive advantage of high neurogenesis is in the low body mass subjects. Several of the effects noted may be related to the peripubertal status of these males, where we lack staging data as well as body composition studies (lean body mass), noting their body weight precedes the rapid gain seen in mid-late adolescence. There is a suggestion that the divergent phenotypes may be more pronounced following ELS, a threat condition which sets the stage for later caloric conservation, but larger *N*'s are required to make a definitive conclusion. Strengths of the study include use of a naturalistic nonhuman primate milieu in contrast to boxed rodents with constant supplies of excess chow and lack of physical activity or phenotypic diversity over the life cycle. Thus, the study may bear more relevance to human neuroplasticity in comparison to rodent studies.

## Figures and Tables

**Table 1:** Predictors of neurogenesis rates (square root doublecortin counts) and rearing effects with post hoc Spearman correlation verification.

	Rearing effect	SQRT DCN	Sp *r*	P
Adolescent incretin (df = 1,11) N = 14				
pGLP-1	F = 0.27; P = 0.61	*F* = 5.53; *P* = 0.04	**.72**	*P* = .003

Adolescent morphometrics (df = 1,11) N = 14				
BMI	F = 1.18; P = 0.30	F = 22.48; P = 0.0006	**−.88**	P = .00002
Circ	F = 0.00; P = 0.98	*F* = 6.80; *P* = 0.02	**−.73**	P = .002
SAD	F = 1.69; P = 0.22	F = 17.56; P = 0.0015	**−.65**	P = .01
Crl	F = 0.20; P = 0.66	F = 3.93; P = 0.07	−.31	P = .27
Body mass	F = 0.20; P = 0.66	F = 16.34; P = 0.0019	**−.74**	P = .002
Age	F = 0.19; P = 0.66	F = 0.58; P = 0.46	−.26	P = .36

Adolescent glucose metabolism (df = 1,8) N = 11				
Insulin (pmol/L)	F = 1.62; P = 0.23	F = 6.50; P = 0.034	**.69 **	P = .017
Glucose (ng/mL)	F = 0.02; P = 0.87	F = 0.29; P = 0.59	−.22	P = .44
IGR	F = 0.82; P = 0.39	F = 4.84; P = 0.058	**.67**	P = .017
HOMA	F = 3.03; P = 0.12	F = 8.77; P = 0.018	**.66**	P = .026

Adolescent lipid profile (mg/dL) (df = 1,11) N = 14				
Triglyc.	F = 0.26; P = 0.61	F = 0.15; P = 0.70	.16	P = .57
HDL	F = 0.16; P = 0.69	F = 4.42; P = 0.059	.32	P = .25
LDL	F = 0.01; P = 0.89	F = 7.15; P = 0.021	**.71**	P = .004
CHOL	F = 0.00; P = 0.95	F = 9.82; P = 0.009	**.67**	P = .008
VLDL	F = 0.02; P = 0.90	F = 2.12; P = 0.17	.36	P = .19

Adult (df = 1,11) N = 14				
Age (years)	F = 2.04; P = 0.18	F = 8.15; P = 0.015	−.32	P = .26
Weight (kg)	F = 0.00; P = 0.99	F = 2.38; P = 0.15	−.43	P = .12

Significant effects bolded. SQRT doublecortin = square root of dentate gyrus immature neurons (mm^3^), Sp *r* = Spearman's rho correlation, pGLP-1 = plasma glucagon-like peptide-1 (pmol/L), Crl = crown-rump length (cm), BMI = body mass index (kg/m^2^), Circ = waist circumference (cm), SAD = sagittal abdominal diameter (cm), IGR = insulin/glucose ratio, HOMA = homeostatic model of assessment, and Triglyc. = triglycerides.

**Table 2:** Comparison of within-rearing group and combined subjects Pearson correlations between adolescent GLP-1 and morphometrics, metabolic markers, and plasma lipids.

Morphometrics	VFD = 10	USC = 4	r-difference	All = 14
BMI (kg)/m^2^	−.81	−.51		−.66
	*P* = .004	*P* = .483	0.61	*P* = .009
Crl (cm)	−.16	.71		−.24
	*P* = .64	*P* = .28	0.34	*P* = .40
Circ (cm)	−.94	.86		−.80
	*P* = .001	*P* = .13	0.017	*P* = .001
SAD (cm)	−.53	.29		−.56
	*P* = .11	*P* = .70	0.42	*P* = .035
Body mass (kg)	−.52	.16		−.51
	*P* = .12	*P* = .83	0.47	*P* = 0.059

Metabolic markers	VFD = 8	USC = 3		All = 11

Insulin^*^ (pmol/L)	.91	.95		.67
	*P* = .002	*P* = .200	0.78	*P* = .023
Glucose (mg/dL)	−.35	−.58		−.43
	*P* = .31	*P* = .41	0.79	*P* = .12
Igr^*^	.87	.99		.71
	*P* = .004	*P* = .054	0.86	*P* = .014
HOMA^*^	.92	.81		.60
	*P* = .001	*P* = .39	0.68	*P* = .049
Euglycemic Cl.^**^ (mg/kg/min)	−.91	−.96		−.88
	*P* = 0.03	.17	0.74	*P* = 0.004

Lipids (mg/dL)	VFD = 10	USC = 4		

Triglycerides	.57	−.30		−.04
	*P* = .079	*P* = .69	0.39	*P* = .87
HDL	.34	−.22		.30
	*P* = .33	*P* = .77	0.60	*P* = .29
LDL	.65	−.65		.65
	*P* = .039	*P* = .34	0.17	*P* = .012
CHOL	.63	−.95		.61
	*P* = .050	*P* = .045	0.039	*P* = .019
VLDL	.63	−.47		.61
	*P* = .047	*P* = .53	0.26	*P* = .019

Adult				
Body mass (kg)	−.71	.98		−.51
	*P* = .021	*P* = .012	0.013	*P* = .062
Age (lunar months)	−.34	−.75		−.49
	*P* = .33	*P* = .24	0.57	*P* = .069

Significant effects bolded. ^*^8 VFD and 3 USC subjects were available for insulin analysis. ^**^5 VFD and 3 USC were available for euglycemic clamp analysis. Crl = crown-rump length, BMI = body mass index, Circ = abdominal circumference (cm), SAD = sagittal abdominal diameter (cm), IGR = insulin/glucose ratio, HOMA = homeostatic model assessment, Euglycemic Cl. = euglycemic insulin clamp procedure, and CHOL = cholesterol.
